# Simultaneous Single-Port Robotic Excision of Diaphragmatic Endometriosis and Mucosa-Sparing Nodulectomy in Rectal Endometriosis

**DOI:** 10.3390/jcm15218274

**Published:** 2026-10-23

**Authors:** Harald Krentel, Petra Klein, Mihai Constantin, Kiyoshi Kanno, Rudy Leon De Wilde, Petya Tanovska

**Affiliations:** 1 Department of Gynecology and Obstetrics, Klinikum Aschaffenburg-Alzenau, 63739 Aschaffenburg, Germany; 2 Department of Gynecology, Kurashiki Medical Center, Kurashiki 710-8522, Japan; 3 School of Medicine and Health Sciences, Carl von Ossietzky University Oldenburg, 26129 Oldenburg, Germany

**Keywords:** endometriosis, rectum, diaphragm, single-port robotics, rectal surgical procedures

## Abstract

The simultaneous appearance of symptomatic severe pelvic and diaphragmatic deep endometriosis represents a particular challenge in surgical treatment of the disease. We report a symptomatic patient with a history of six previous laparoscopic surgeries for endometriosis who underwent a single-port robotic-assisted complete excision of histopathologically confirmed endometriosis with combined mucosa-sparing rectal nodulectomy and full-thickness resection of the central right diaphragm, performed through a single 3 cm supraumbilical incision. The single-port robotic approach, recently introduced into gynecological minimally invasive surgery, enabled resection of two anatomically distant disease sites—the deep pelvis and the right hemidiaphragm—through one access port without repositioning of the port site. To the best of our knowledge, this is the first case report on simultaneous single-port robotic excision of diaphragmatic and rectal deep endometriosis.

## 1. Introduction

Deep endometriosis (DE) of the diaphragm is a rare but clinically important manifestation of extrapelvic endometriosis, when related to symptoms like catamenial right shoulder, scapular, or chest pain or catamenial pneumothorax. Robotic-assisted multiport surgery has been increasingly adopted for the surgical treatment of symptomatic diaphragmatic endometriosis [1]. In the largest published series to date, robotic excision of severe diaphragmatic endometriosis was performed in 60 consecutive patients, with complete relief of disease-related symptoms reported in the majority of cases, supporting the role of radical excision over ablation or fenestration alone in experienced, multidisciplinary centers [2]. The surgical strategy for rectal DE is guided by localization and number of nodules, nodule size and circumferential extent, ranging from mucosa-sparing nodulectomy to full-thickness disc excision or segmental resection. The concept of mucosa-sparing nodulectomy combines the complete excision of the rectal disease without entering the bowel lumen [3]. Single-port (SP) robotic platforms (da Vinci SP, Intuitive Surgical, Sunnyvale, CA, USA) have recently been introduced to minimally invasive gynecologic surgery, and first experiences in benign indications have been reported [4,5]. Combined single-port robotic resection of diaphragmatic and rectal endometriosis in a single operative session has, to our knowledge, not been previously reported.

## 2. Case Presentation

The 31-year-old patient presented to our endometriosis center with recurrent symptoms after six previous laparoscopic surgeries for endometriosis, including rectal shaving, neurolysis of the lumbosacral plexus and ablation of right-sided diaphragmatic endometriosis during three previous procedures. The main symptoms at the time of presentation were pain in the middle and right upper abdomen and a feeling of restricted breathing, dyschezia, bloating and right-sided pelvic pain (7 NAS). Different medical treatment approaches were not successful or caused side effects over time, and additionally a focal nodular hyperplasia of the liver limited hormonal options. On clinical examination, no deep infiltrating endometriosis was palpable. Systematic transvaginal ultrasound revealed a painful 14 mm endometriosis nodule of the anterior rectal wall at approximately 12 cm from the anal verge, which was classified #Enzian (u) C2. The rectal sliding sign was negative. On transabdominal ultrasound, both kidneys were normal. The upper liver margin and the pleura could be visualized, but possible deep endometriosis lesions could not be assessed reliably by ultrasound. Presurgical T2-weighted magnetic resonance imaging showed a 6 mm hyperintense cystic lesion suspicious for right-sided deep diaphragmatic endometriosis.

**Figure 1:**
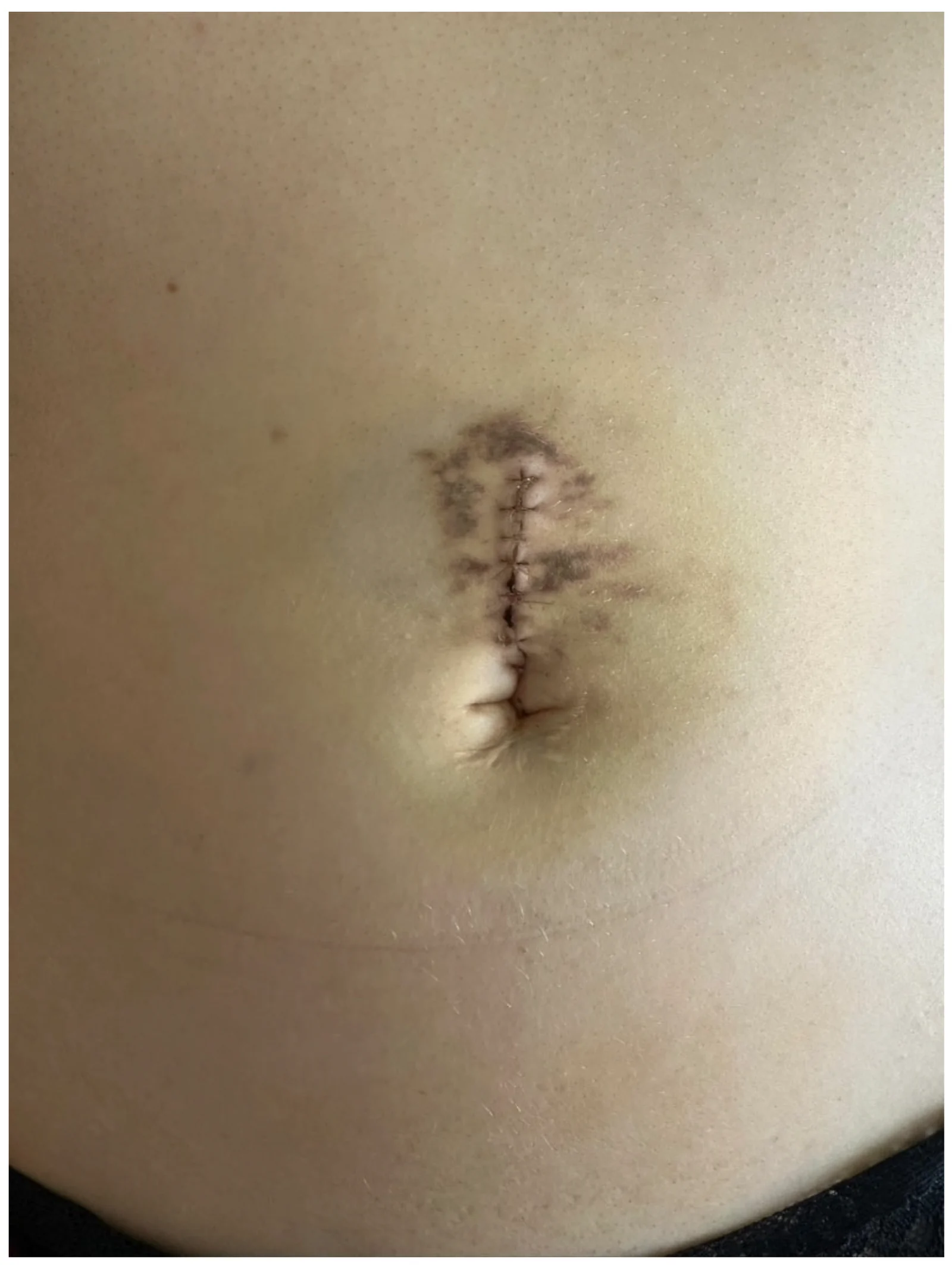
Supraumbilical incision at day 2 after surgery.

We performed a single-port robot-assisted complete excision of diaphragmatic and rectal endometriosis using the DaVinci SP system (Intuitive Surgical, Sunnyvale, CA, USA). In order to guarantee adequate working distance and instrument reach to both the pelvis (rectum) and the right hemidiaphragm, we decided to use a supraumbilical approach (Figure 1). A single multichannel robotic port (da Vinci SP access port) was inserted through a 3 cm incision. We used a pneumoperitoneum with intrabdominal pressure of 12 mmHg. The first part of the surgery was performed with the patient in left-sided reverse Trendelenburg position.

**Figure 2:**
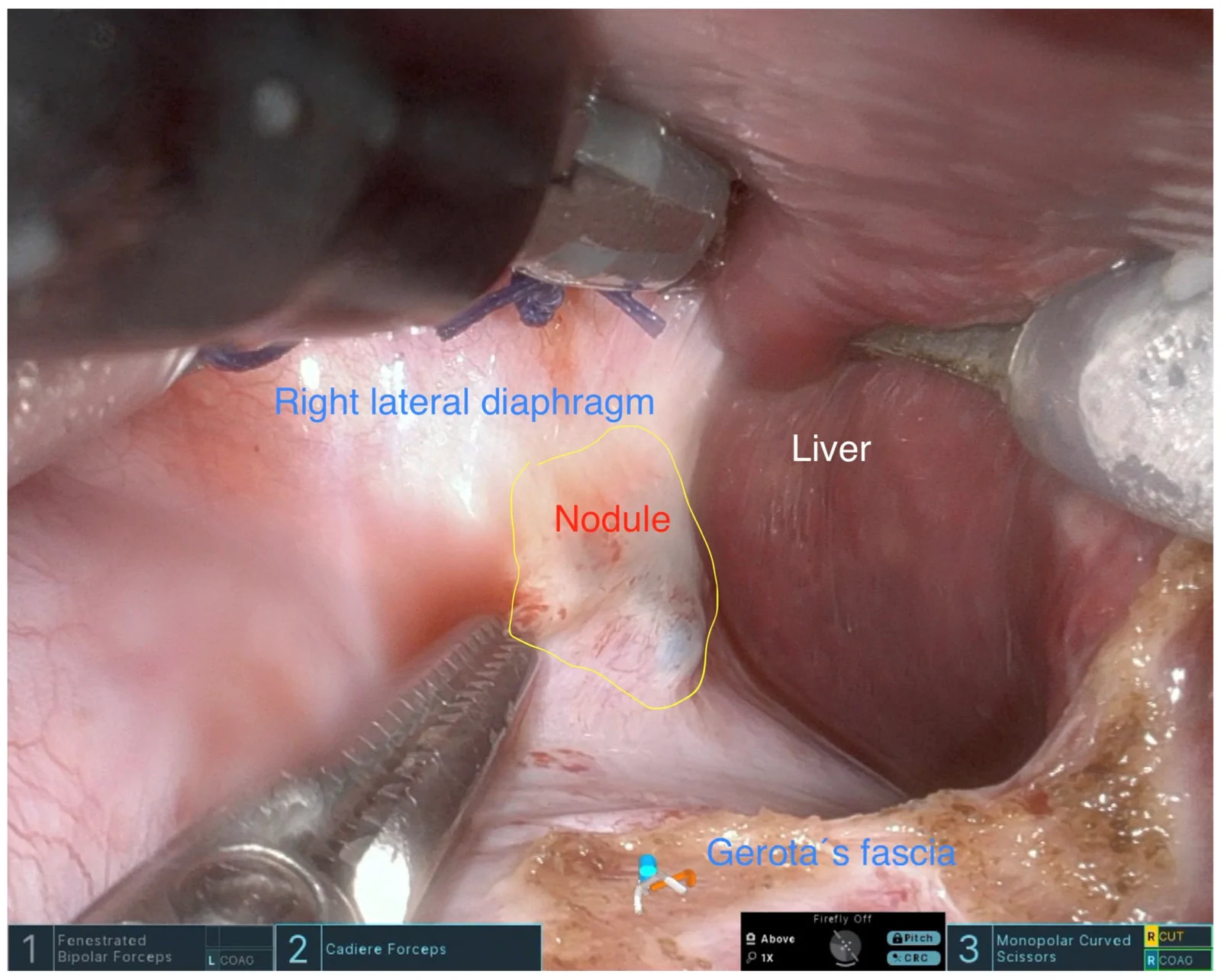
Deep endometriosis nodule of the lateral right diaphragm.

**Figure 3:**
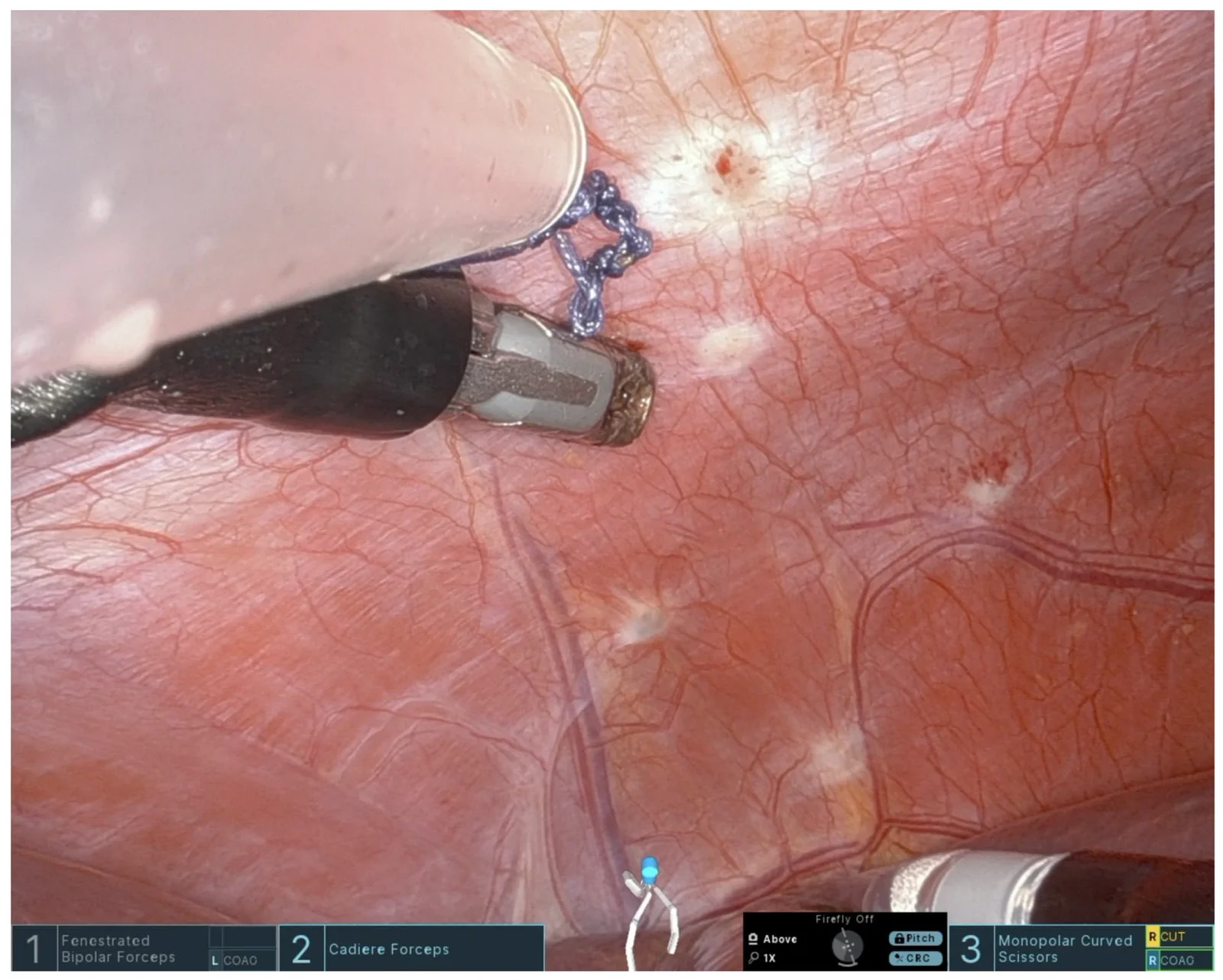
Multiple endometriosis lesions of the right diaphragm.

**Figure 4:**
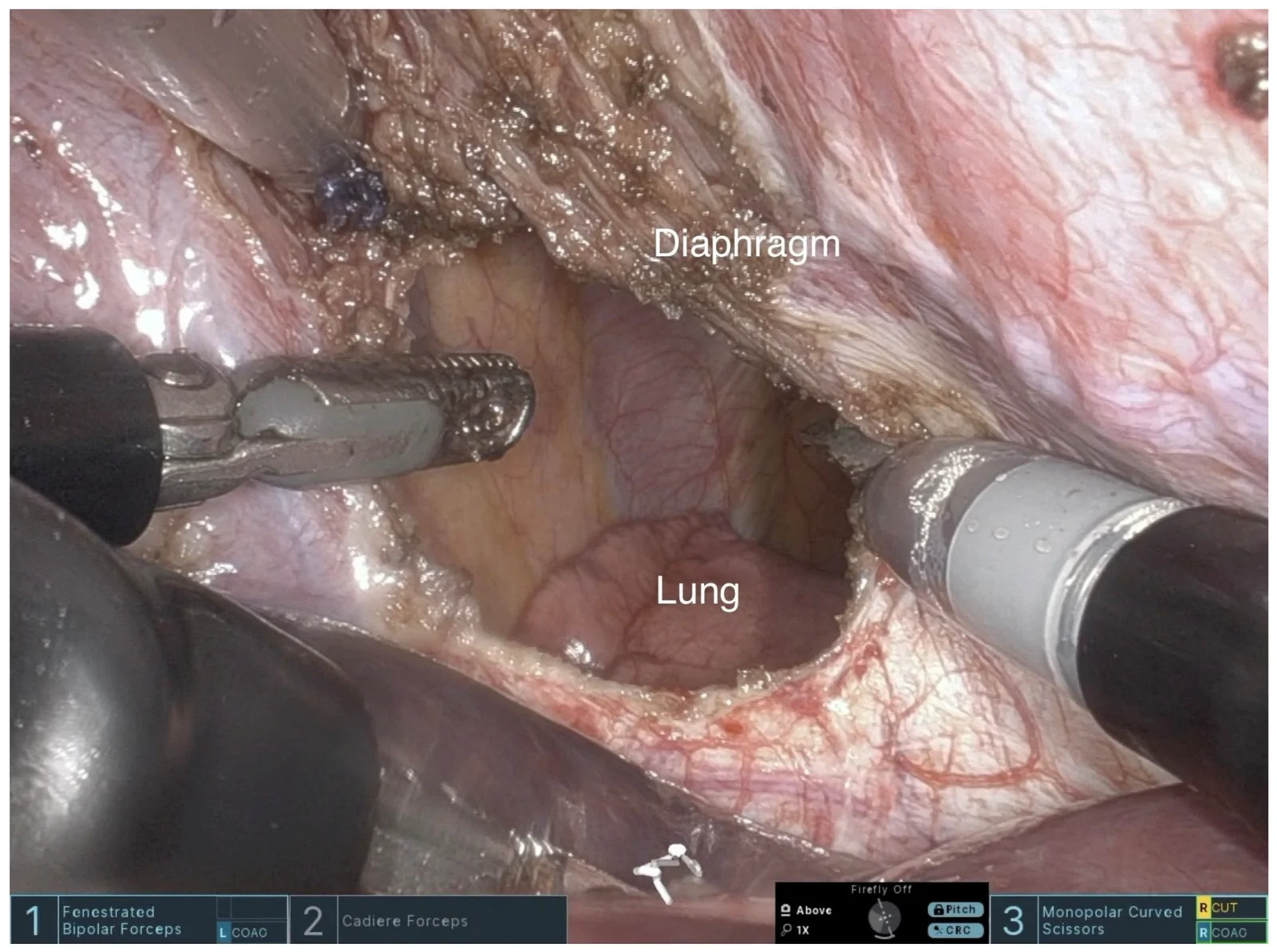
View through the diaphragmatic defect after full-thickness resection, showing the underlying lung.

After liver mobilization, we excised deep endometriosis from the peritoneum and the retroperitoneal tissue posterior to the liver and cranial to the right kidney (Gerota’s fascia) and subsequently performed a full-thickness diaphragmatic and pleural resection with several nodules in one specimen of 3.5 × 2.5 × 0.4 cm. After opening of the pleura, the surgical site was inspected for additional thoracic lesions. Pleura and diaphragm were sutured with continuous barbed double-layer suture (V-Loc, 3-0, Medtronic, Dublin, Irland) and additional non-reabsorbable figure-of-eight sutures (Figure 2, Figure 3 and Figure 4). Additional small superficial lesions could be excised without further need for suturing. Lung re-expansion was confirmed after placement of a temporary chest drain that was necessary in this case. As diaphragmatic resection was planned, a double-lumen endotracheal tube was used for intubation.

**Figure 5:**
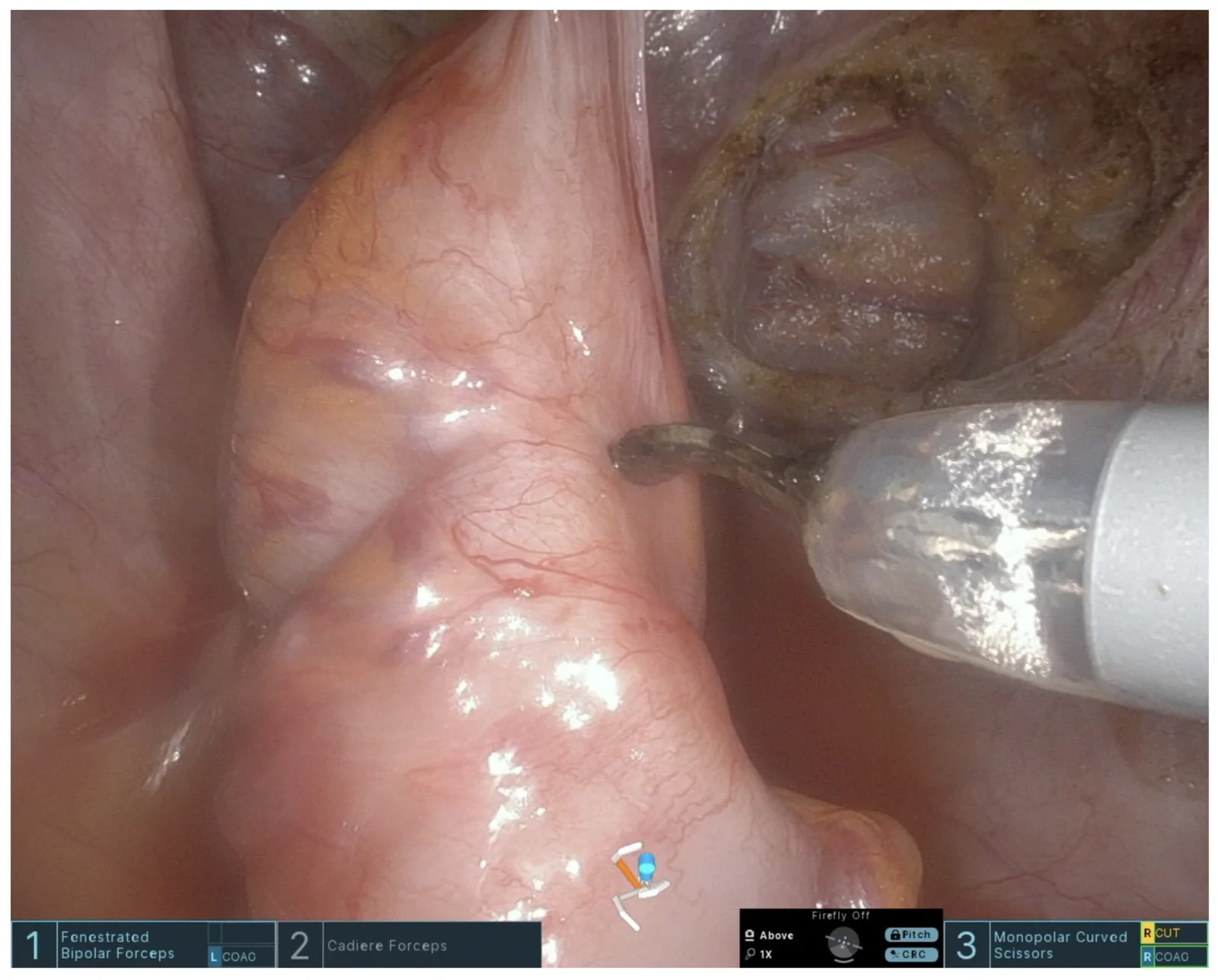
Rectal wall with almost invisible but palpable endometriosis nodule prior to excision.

**Figure 6:**
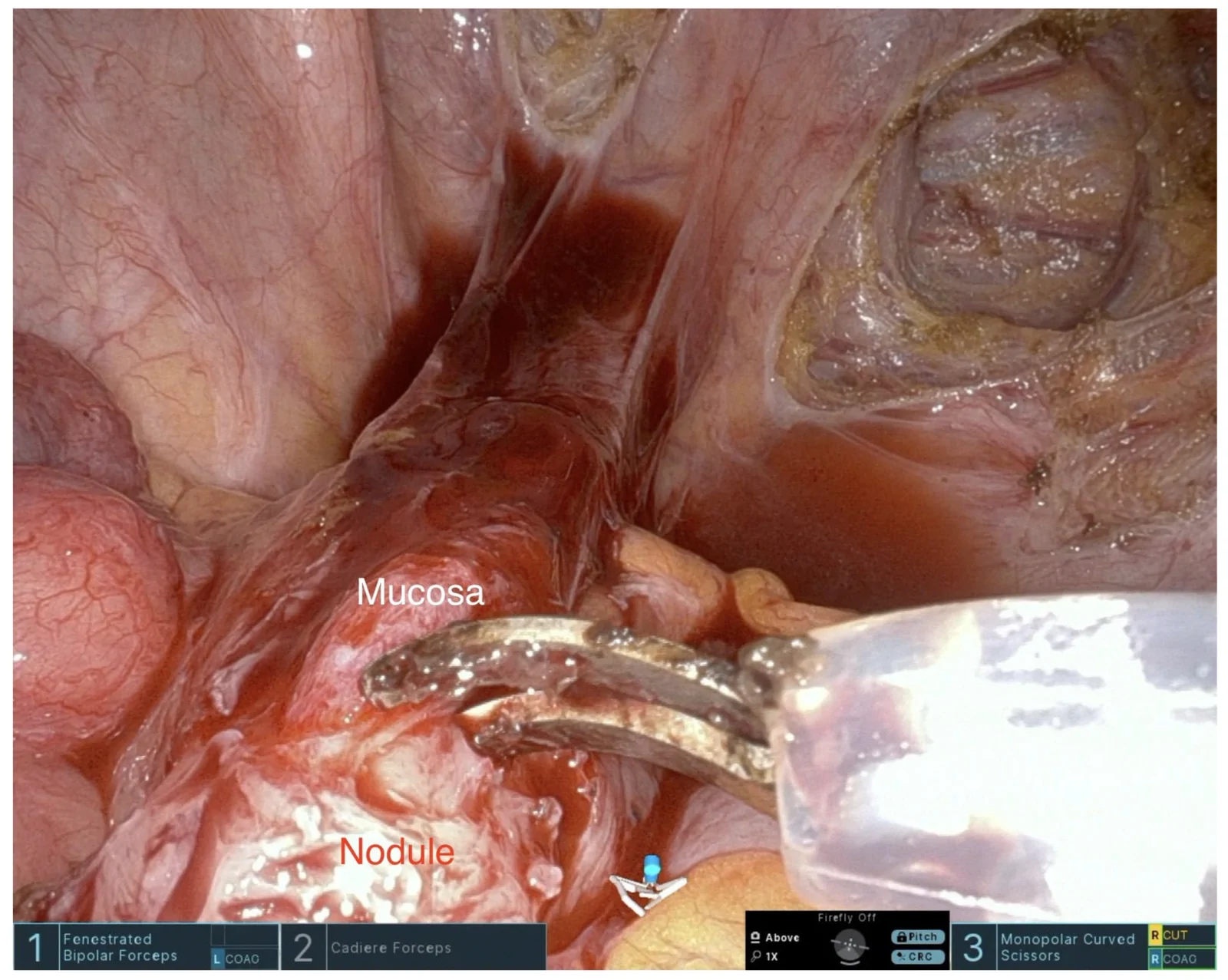
Excision of the rectal endometriosis nodule with sparing of the mucosa using cold scissors.

**Figure 7:**
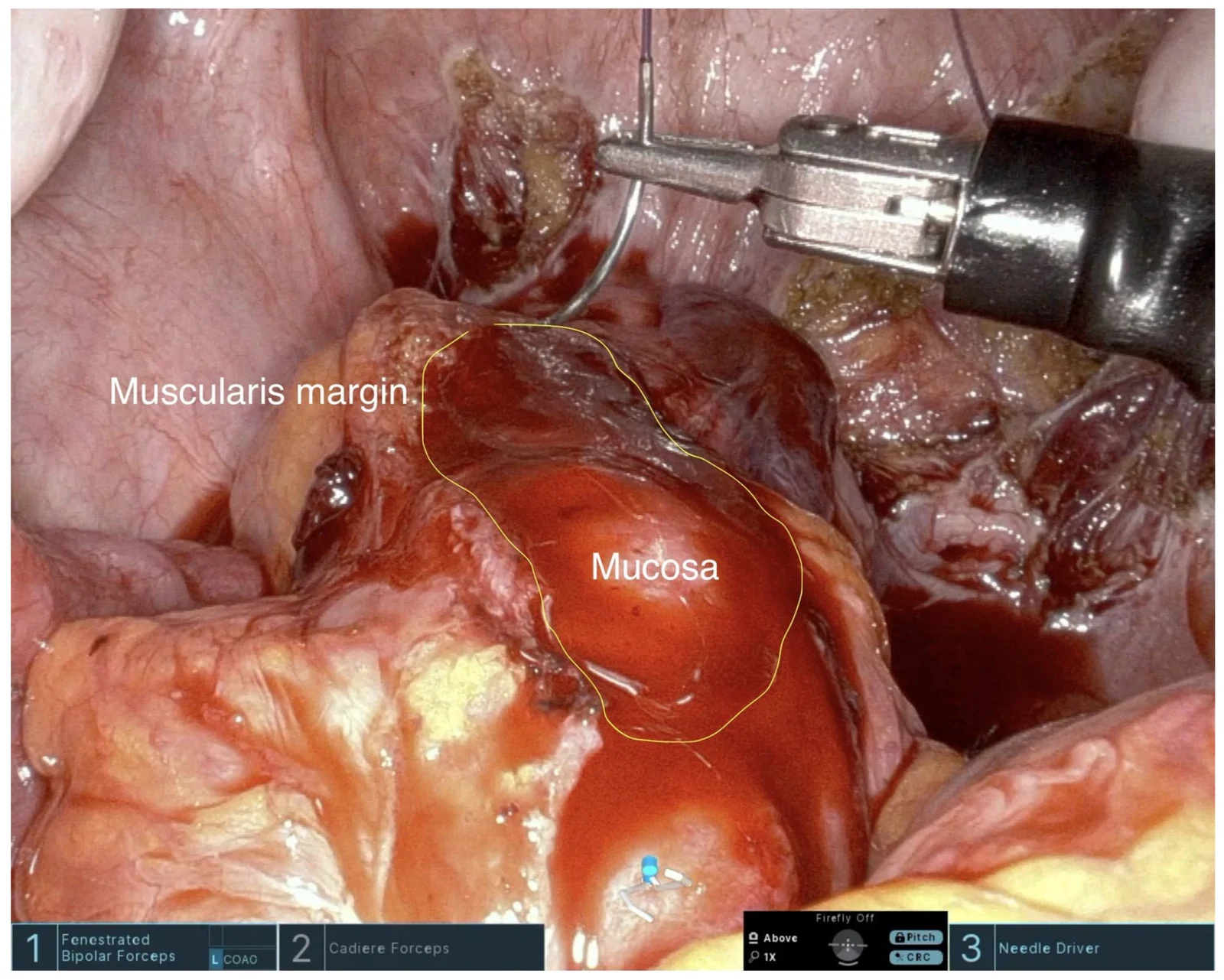
Muscularis margin and spared mucosa exposed with transanal air insufflation after complete excision of the rectal endometriosis nodule.

**Figure 8:**
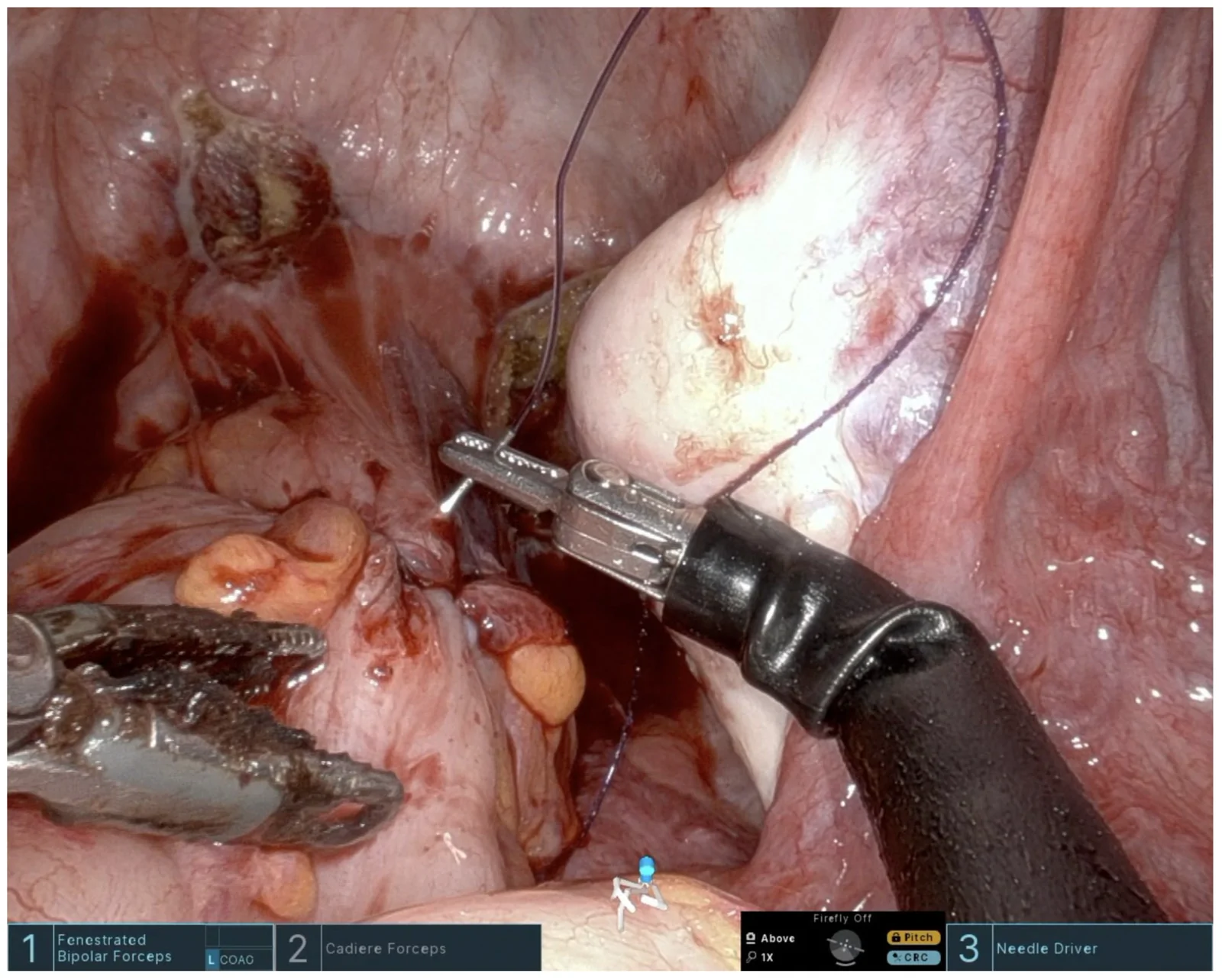
Continuous barbed suture (3-0) closure of the anterior muscular and serosal layer of the rectal wall.

The position of the patient was then changed to Trendelenburg, and we continued the surgery with complete excision of pelvic endometriosis including a right-sided pararectal nodule. Mucosa-sparing rectal nodulectomy was performed using cold scissors. After complete excision of the nodule, we inserted air through a transanal probe in order to create the so-called “bubble sign” of the mucosa and performed a continuous transversal single-layer barbed suture (V-Loc, 3-0, Medtronic, Minneapolis, MN, USA) of the rectal muscularis and serosa (Figure 5, Figure 6, Figure 7 and Figure 8). At the end of the procedure, the air leak test confirmed mucosal integrity. A colorectal surgeon was on stand-by but not directly involved, while a thoracic surgeon joined the team for supervision and guidance.

The disease was classified as #ENZIAN (s) P2 B0/2 C2 F (right diaphragm). Histopathology confirmed rectal endometriosis within the excised rectal muscular layer with a diameter of 1.5 cm, and endometriosis and fibrosis within the 3.5 × 2.5 × 0.4 cm specimen of the right diaphragm. Console time was 232 min and total skin-to-skin time was 283 min. Blood loss was minimal and no complication occurred. Postoperative course was uneventful and the patient was discharged on day 3 after surgery. The patient was contacted by phone six weeks and three months after surgery. Recovery was uneventful and the patient reported constant improvement. After three months, the pelvic pain decreased from 7 to 3 (NAS) with normal bowel and bladder function. Right-sided upper abdominal and thoracic pain disappeared after surgery, while a retrosternal discomfort was still persistent at 3-month follow-up. Further long-term follow-up is scheduled at 6, 12 and 24 months. A literature search of PubMed/MEDLINE and PubMed Central was performed in September 2026 using the terms “single-port,” “robotic,” and “diaphragmatic endometriosis” to identify previously published reports. To our knowledge, only one prior report on single-port robotic-assisted diaphragmatic endometriosis has been identified, published as a congress abstract, with no full peer-reviewed article available to date [6].

## 3. Discussion

This case illustrates, to our knowledge, the first reported use of DaVinci SP for a combined excision of diaphragmatic endometriosis and rectal endometriosis in one operative session. Single-port robotic endometriosis surgery has typically used a transumbilical or infraumbilical incision, which provides excellent access to the pelvis but may limit the reach to the upper abdomen [7]. In our case, a supraumbilical midline access, positioned cranial to the umbilicus, was chosen specifically to balance reach to both the pelvis and the right hemidiaphragm from a single position. We consider this single, cranialized midline access a reasonable compromise for combined procedures, although the optimal port position for combined pelvic/upper-abdominal single-port surgery has not yet been systematically studied and may need to be individualized according to patient height and the relative extent of pelvic versus diaphragmatic disease. Whether to combine resection of pelvic and diaphragmatic endometriosis in a single procedure or to stage the interventions remains a matter of surgical judgment under consideration of feasibility and possible complications. We elected to proceed in a single stage, as presurgical transvaginal ultrasound revealed an #Enzian C2 rectal lesion, feasible for indicating mucosa-sparing nodulectomy and thus avoiding the opening of the bowel lumen. This technique has recently been described and analyzed elsewhere [3]. A single-stage multiport combined approach has previously been reported for diaphragmatic disease in combination with rectal disease, supporting the feasibility of addressing multiple disease compartments robotically within one session in appropriately selected patients and centers with adequate multidisciplinary support (including thoracic surgery availability) [8]. However, avoiding entry into the bowel lumen in our case further reduced the theoretical risk of intraperitoneal contamination and anastomotic-type complications, which is of particular relevance when combined with a synchronous diaphragmatic procedure that already carries its own risk profile.

This case report also highlights the well-known diagnostic challenges in diaphragmatic endometriosis. Presurgical MR imaging underestimates the disease and only revealed one 6 mm hyperintense lesion. Adequate exposure of the right hemidiaphragm, particularly its posterior aspect, requires mobilization of the liver. Division of the right triangular and falciform ligaments to allow caudal retraction of the liver is consistently described as a critical preparatory step in robotic and laparoscopic diaphragmatic endometriosis surgery, and incomplete liver mobilization is a recognized cause of incomplete lesion excision or missed posterior lesions [9]. In the single-port setting, this step is rendered technically demanding by the reduced instrument spread compared with multiport platforms. Nonetheless, the wristed single-port instruments with distal triangulation for precise maneuvers in narrow spaces, the relocate mode and the camera’s distal articulation (cobra position) allowed adequate retraction and visualization in this case. If necessary, an additional trocar can be placed at any time during surgery to allow the introduction of assisting laparoscopic instruments. Due to the single arm setting, access to the patient and the abdominal wall is less limited compared to multiport procedures. Another advantage of the SP technique is the very quick re-docking whenever changes in patient positioning are necessary during surgery. Additionally, resected tissue can be easily removed from the abdominal cavity through the single incision.

Strengths and limitations: This report describes a single case experience and therefore cannot establish generalizable safety or efficacy data for the single-port approach to combined diaphragmatic and rectal endometriosis. Operative time, cost, and the learning curve associated with single-port instrumentation in this anatomically demanding combination of procedures require further evaluation in larger series. However, the present case demonstrates the feasibility of true multi-quadrant surgery using the da Vinci SP platform through a single access port. A supraumbilical approach allowed safe access to both the right hemidiaphragm and the deep pelvis without the need for additional ports or repositioning of the access site. This finding highlights a unique technical advantage of the SP platform over conventional multiport robotic systems, particularly for procedures requiring access to anatomically distant operative fields.

## 4. Conclusions

Simultaneous single-port robotic excision of diaphragmatic endometriosis and mucosa-sparing rectal nodulectomy is technically feasible in carefully selected patients treated in a multidisciplinary, high-volume endometriosis center. This case extends the application of single-port robotic technology beyond isolated pelvic endometriosis surgery to combined extrapelvic and pelvic disease, and may serve as a technical reference for centers considering this approach. Further cases and comparative studies are needed to define patient selection criteria, optimal port placement, and outcomes relative to multiport robotic and conventional laparoscopic techniques.

## Data Availability

The data presented in this case report are available on request from the corresponding author, in accordance with patient privacy considerations.

## References

[1] Naem A., Roman H., Martin D.C., Krentel H. (2024). A Bird-Eye View of Diaphragmatic Endometriosis: Current Practices and Future Perspectives. Front. Med..

[2] D’Ancona G., Merlot B., Chanavaz-Lacheray I., Braund S., Kade S., Dennis T., Roman H. (2024). Robotic-Assisted Laparoscopy Excision of a Severe Form of Diaphragmatic Endometriosis: A Retrospective Study of 60 Patients. J. Minim. Invasive Gynecol..

[3] Krentel H., Naem A., Andrikos A., Otto K., Schimmack S., De Wilde R.L., Keckstein J., Tanovska P., Andrikos D. (2026). Postoperative and Long-Term Outcomes of Nerve-Sparing Segmental Rectal Resection and Complete Nodular Resection of Rectal Endometriosis. Facts Views Vis. Obgyn.

[4] Alwafai Z., Schweder E., Poschkamp B., Langheinrich M., Kersting S., Hummel R., Zygmunt M. (2025). Single-Port Robotic Surgery in Gynecology: First Experience in German-Speaking Countries Using the Da Vinci SP System in Benign and Neoplastic Diseases. J. Robot. Surg..

[5] Kanno K., Higuchi N., Masuda S., Onji H., Taniguchi R., Ochi Y., Kurose Y., Sawada M., Yanai S., Hoshiba T. (2025). Fluorescence-Guided Nerve-Sparing Surgery for Deep Endometriosis Using Indocyanine Green. Facts Views Vis. Obgyn.

[6] Kohn J.R., Yang Q., Ripley R.T., Guan X. (2025). Excision of Diaphragmatic Endometriosis Using the Robotic Single-Port System (Da Vinci SP). J. Minim. Invasive Gynecol..

[7] Kanno K., Andou M., Sawada M., Yanai S. (2025). Segmental Bowel Resection for Rectal Endometriosis Using the da Vinci SP. J. Minim. Invasive Gynecol..

[8] Krentel H., Burla L., Tanovska P., Klein P., Hoche C., Constantin M., de Wilde M.S., Devassy R., Keckstein J., Vlachodimitris I. (2026). Robotic-Assisted Compared to Conventional Laparoscopic Surgery for Colorectal Endometriosis: Perioperative Outcomes in the Context of #Enzian-Defined Anatomical Complexity. J. Robot. Surg..

[9] Roman H., Dennis T., Grigoriadis G., Merlot B. (2022). Robotic Management of Diaphragmatic Endometriosis in 10 Steps. J. Minim. Invasive Gynecol..

